# Salivary pellicle modulates biofilm formation on titanium surfaces

**DOI:** 10.1007/s00784-023-05230-9

**Published:** 2023-08-30

**Authors:** Miryam Martínez-Hernández, Juan Pablo Reyes-Grajeda, Matthias Hannig, Argelia Almaguer-Flores

**Affiliations:** 1 Laboratorio de Biointerfases, División de Estudios de Posgrado e Investigación, Facultad de Odontología, Universidad Nacional Autónoma de México, Circuito Exterior s/n, Ciudad Universitaria, 04510 Mexico City, Mexico; 2 Laboratorio de Estructura de Proteínas, Instituto Nacional de Medicina Genómica (INMEGEN), 14610 Mexico City, Mexico; 3 Clinic of Operative Dentistry, Periodontology and Preventive Dentistry, University Hospital, Saarland University, Building 73, 66421 Homburg, Saarland Germany

**Keywords:** Salivary pellicle, Titanium surfaces, Dental implants, Dental enamel, Oral bacteria, Biofilm formation

## Abstract

**Objectives:**

The present study aimed to evaluate the potential of the salivary pellicle (SP) formed on titanium (Ti) surfaces to modulate the formation of a biofilm composed of *Streptococcus gordonii*, *Actinomyces naeslundii*, *Fusobacterium nucleatum*, and *Porphyromonas gingivalis*.

**Materials and methods:**

Ti substrates were incubated for 2 h with a pool of saliva samples obtained from 10 systemically and periodontally healthy subjects. Enamel substrates were included as a biological reference. Scanning electron microscopy (SEM) and Raman spectroscopy analysis were used to analyze the formation of the salivary pellicle. After the SP formation, the surfaces were incubated for 12 h with a mix of *Streptococcus gordonii*, *Actinomyces naeslundii*, *Fusobacterium nucleatum*, and *Porphyromonas gingivalis*. The number of bacterial cells attached to each surface was determined by the XTT assay while bacterial viability was analyzed by fluorescence microscopy using the LIVE/DEAD® BacLight^TM^ kit.

**Results:**

The SEM and Raman spectroscopy analysis confirmed the presence of a salivary pellicle formed on the tested surfaces. Regarding the biofilm formation, the presence of the SP decreases the number of the bacterial cells detected in the test surfaces, compared with the uncover substrates. Even more, the SP-covered substrates showed similar bacterial counts in both Ti and enamel surfaces, meaning that the physicochemical differences of the substrates were less determinant than the presence of the SP. While on the SP-uncover substrates, differences in the bacterial adhesion patterns were directly related to the physicochemical nature of the substrates.

**Conclusions:**

The salivary pellicle was the main modulator in the development of the biofilm consisting of representative oral bacteria on the Ti substrates.

**Clinical relevance:**

The results of this study provide valuable information on the modulatory effect of the salivary pellicle on biofilm formation; such information allows us to understand better the events involved in the formation of oral biofilms on Ti dental implants.

**Supplementary Information:**

The online version contains supplementary material available at 10.1007/s00784-023-05230-9.

## Introduction

Within the oral cavity, where a variety of natural and artificial surfaces are colonized with complex biofilms, saliva is essential for maintaining homeostasis [1]. Besides its function ranging from digestion to lubrication, remineralization, and beyond [2], saliva is implicated in the formation of a protein layer termed salivary pellicle (SP), formed by the adsorption of salivary components onto surfaces such as natural dentition and dental biomaterials [3]. SP contributes to basic outcomes in dentistry, including the protection of oral surfaces against wear [4], modulation of periodontal wound healing [5], and bacterial adhesion on oral surfaces [6]. Currently, dental materials have been less researched than natural teeth in terms of SP formation [7], and it is known that the physicochemical characteristics of these biomaterials influence the composition and structure of the SP [8]. Different types of biomaterials can be placed in the oral cavity, including titanium dental implants. Dental implant therapy is a predictable therapeutic option for rehabilitating partially or fully edentulous patients, providing long-term function (10 years plus) and esthetics [9]. However, due to the abundance of microorganisms in the oral cavity, dental implants are susceptible to contamination [10], which can result in biological complications, including implant failure [11].

Once a clean implant abutment surface is exposed to the oral cavity, it is immediately covered by a salivary pellicle [12], which in turn significantly influences microbial adhesion to the Ti substratum [13, 14]. It has been reported that bacteria colonize Ti surfaces as quickly as within 30 min after dental implant placement [15] and can form a complex biofilm within a couple of weeks [16]. In this regard, there is extensive evidence that biofilm accumulation at the implant-abutment interface can result in inflammatory cell infiltration, ultimately leading to bone loss [17].

In light of the fundamental role that SP plays in peri-implant health, our group previously reported that the presence of saliva inhibited the adhesion of representative oral bacteria to titanium films [18]. To deepen the understanding of the effect of salivary conditioning of titanium surfaces on the adhesion of oral bacteria, in another study by our group, different microstructured titanium substrates were incubated intraorally on periodontally healthy and periodontitis subjects, finding that the periodontal status, and therefore the salivary composition and microbiota of the volunteers who participated in this study, was more determinant in modulating the identity and proportions of oral species within biofilms formed on these substrates than the surface characteristics of Ti surfaces itself [19]. Therefore, while the formation of the oral biofilm on the microstructured Ti surfaces did not depend directly on the characteristics of the substrate as shown from the previous study [19], the amount and identity of the proteins forming part of the salivary film did depend on the physicochemical properties (e.g., roughness and wettability) of the substrates [20]. In combination, these observations support the assertion that saliva/salivary pellicle–titanium surface interactions assist in the development of oral biofilms on titanium dental implants.

Studying the acquired salivary film process is important because it connects artificial and tooth surfaces with the oral environment, as well as the fact it contributes significantly to the development of severe oral diseases, including erosion, dental caries, periodontal disease, and peri-implant infection [21]. With the growing use of dental implant restorations, studies exploring SP formation on titanium substrates have become increasingly important [22, 23]. Therefore, detailed knowledge of the biological interactions occurring at the implant interface with host fluids is necessary. The present study examines how the SP modulates the formation of a biofilm constituted by *Streptococcus gordonii*, *Actinomyces naeslundii*, *Fusobacterium nucleatum*, and *Porphyromonas gingivalis* on Ti surfaces.

## Materials and methods

### Tested surfaces

Ti disks with diameters of 15 mm were prepared from 1-mm-thick sheets of grade 2 unalloyed Ti (ASTM F67). The methods used to produce the pretreated Ti surfaces have been previously described [20]. The Ti surfaces are relatively smooth with an average roughness of (Ra) < 0.44 μm and a water contact angle (WCA) of 91.5 ± 0.5°. All Ti disks were fabricated by the Institut Straumann AG (Basel, Switzerland) and were shipped to us ready for use. Natural enamel surfaces were compassed as biological reference substrates (Ra < 0.05 and WCA of 44.1 ± 0.3°). Round enamel surfaces of 6 mm diameter were obtained from the buccal face of cattle incisors, the enamel surfaces were prepared and cleaned by following previously described protocols [24, 25]. In each experiment, four Ti disks and 25 enamel surfaces were used to normalize the total area evaluated (8 cm^2^). All experiments were repeated at least three times.

### Ethics statements, saliva collection, and salivary pellicle formation assays

Whole saliva samples were collected from 10 healthy volunteers (5 females and 5 males), from 28 to 42 years old, without any active carious lesions or history of periodontal disease. Donors consented to saliva sample collection and analysis by signing an informed consent approved by the Ethics Committee for Human Studies of the Division of Postgraduate Studies and Research, School of Dentistry, National Autonomous University of Mexico (CIE/0708/11/2018), which was performed in accordance with the Helsinki Declaration.

All the saliva donors were currently nonsmokers who did not receive any form of periodontal therapy other than professional supragingival plaque removal in the past. Volunteers had at least 20 natural teeth (excluding third molars). Exclusion criteria included pregnancy, nursing women, antibiotic therapy within the previous 3 months, and any systemic condition that could influence the course of periodontal disease (e.g., diabetes, human immunodeficiency virus/acquired immunodeficiency syndrome, or autoimmune diseases). Clinical measurements were taken from each subject at six sites per tooth (mesiobuccal, buccal, distobuccal, distolingual, lingual, and mesiolingual) at all teeth excluding third molars (a maximum of 168 sites per subject) as previously described [19].

Before salivary collection, subjects were asked to rinse the mouth with clean water for 30 s to remove desquamated epithelial cells, microorganisms, and food remnants. After the mouth rinse, subjects were asked to wait for a minute before collection. A 50-mL sterile tube was used to collect passive drooled saliva for 3 min. The tube was maintained on ice during collection to ensure the integrity of the sample. The saliva samples were centrifuged at 15,000g, 4 °C for 15 min; the resulting cleared supernatants supplemented with the EDTA-free complete protease inhibitor mix (Roche Diagnostics, Penzberg, Germany) were then directly frozen at −80 °C until further assays.

### Protein integrity and 2D mapping of saliva

To evaluate the integrity and the intraindividual variability between the saliva samples from the volunteers, the individual saliva samples were characterized by means of a 1-dimensional polyacrylamide (1D-PAGE), while a pool of the saliva samples was analyzed by 2-dimensional polyacrylamide gels. The protein concentration in the samples was determined by BCA assay. The pool of saliva samples was used for the in vitro salivary pellicle formation on the tested surfaces.

Briefly, solubilized samples (5–20 μL) were loaded manually onto 4% EF strips (GE, Healthcare) with a pH range of 3–10 and 4–7. Proteins were focused in the first electrophoretic dimension for 25,000 Vh. Each focused IEF gel was placed on top of a polymerized slab gel and held in place with 1% agarose. Second-dimensional slab gels were resolved in the Mr range between ca. 10 and 250 kDa using a Mini-PROTEAN® Tetra Cell (1300 Vh, 20 °C). Electrophoresed gels were fixed and stained in a Coomassie Brilliant Blue G-250 staining solution and digitalized using a Bio-Rad ChemiDoc Imaging Systems.

### In vitro salivary pellicle formation

Tested surfaces, Ti and enamel, were individually plated in 24-well plates with 400 μL of a pool of the saliva samples and incubated for 2 h at 37 °C under constant shaking. After incubation, the surfaces were washed twice with 1 mL of H_2_O_dd_ to remove non-adsorbed proteins. The amount of the protein adsorbed on the tested substrates was determined by BCA assay. To confirm the formation of the salivary pellicle, the saliva-coated surfaces were evaluated by using scanning electron microscopy (SEM) (Tescan VEGA3); the images were obtained in secondary electron mode at different magnifications and by Raman spectroscopy (Senterra Raman microscope equipped with 532-nm laser [Bruker Optics, Ettlingen, Germany]).

In addition, another set of samples was used for the transmission electron microscopy (TEM) confirmation of the in situ salivary pellicle formation on the tested substrates, for which a removable acrylic device with enamel or Ti surfaces placed in the buccal area was worn for 3 min and 2-h (Supplementary information).

### Biofilm development

The oral anaerobic bacteria strains *S. gordonii* (ATCC 10558), *A. naeslundii* (ATCC 12104), *F. nucleatum* subsp. *nucleatum* (ATCC 25586), and *P. gingivalis* (ATCC 33277) were used for in vitro biofilm formation assays on the tested surfaces. All strains were obtained as lyophilized cultures from the American Type Culture Collection (ATCC, Rockville, MD, USA). The strains were rehydrated as previously described [26]. After the in vitro formation of the salivary pellicles on the test surfaces, the pellicle-coated surfaces were added with a pool of the four bacterial strains (1 × 10^6^ cells of each bacterial species). Then, bacterial-supplemented surfaces were incubated for 12 h at 35 °C under anaerobic conditions using enriched Mycoplasma broth media (5μg/mL hemin and 0.3 μg/mL menadione). After anaerobic incubation, each surface was washed twice with 1 mL of enriched broth to detach bacterial cells not adhered to the surfaces. The viability of bacterial cells adhered to each test surface was analyzed using the XTT kit (Sigma-Aldrich Corp., St Louis., MO, USA). Briefly, the washed surfaces were transferred to the immediate well where they were added with 1 mL of enriched broth to be sonicated for 5 periods of 10 s each, with the aim of detaching the bacteria adhered to each set of test surfaces, the detached bacteria were incubated with 50 μL of 5% XTT. The absorbance was analyzed at a wavelength of 450 nm using a FilterMax F5 equipment.

Another set of samples was processed to evaluate bacterial adhesion on each test substrate by fluorescence microscopy. Briefly, after the incubation with the bacterial suspension, each surface was washed three times with H_2_O_dd_, and the samples were fixed to a slide where 5 μL of LIVE/DEAD® BacLight^TM^ stain (Invitrogen, Molecular Probes, Carlsbad, CA, USA) was added, the samples were incubated for 20–30 min. in a humid chamber, after the incubation time the samples were washed with abundant H_2_O_dd_ and then were immediately observed under a epifluorescence microscopy (Axioskop II, Zeiss, Oberkochen, Germany).

### Statistical analysis

Data from the quantity of adsorbed proteins and the total bacterial counts are presented as the mean ± standard error of the mean (SEM). Data were analyzed using analysis of variance (ANOVA) followed by Bonferroni’s correction for multiple comparisons, significant differences were determined.

## Results

In the present work, the potential of the salivary pellicle (SP), formed on titanium (Ti) and dental enamel surfaces, to modulate the initial biofilm formation constituted by *S. gordonii*, *A. naeslundii*, *F. nucleatum*, and *P. gingivalis* biofilm formation was investigated.

### Saliva collection and pellicle formation assays

**Fig. 1:**
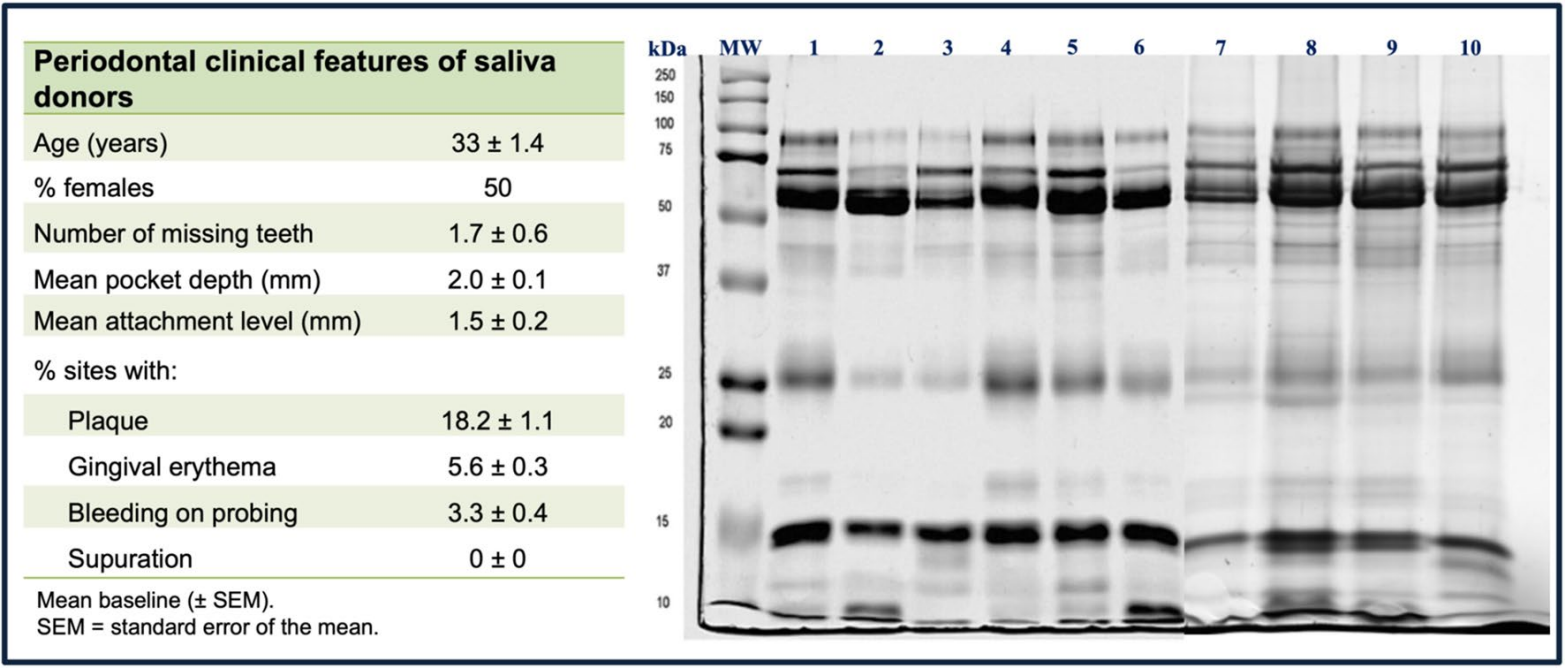
The table on the left shows the periodontal clinical characteristics of the saliva donors. The image on the right displays the representative SDS polyacrylamide slab gel analysis of whole saliva samples stained with Coomassie blue, where the separation pattern and content of different salivary proteins between the saliva donors are shown

Ten periodontally healthy subjects (periodontal health defined as the absence of sites with probing depths of ≤ 3 mm with full-mouth bleeding on probing < 10%) [27] were selected for the whole saliva collection. The clinical characteristics of the saliva donors and the protein profile of each sample collected are shown in Fig. 1.

**Fig. 2:**
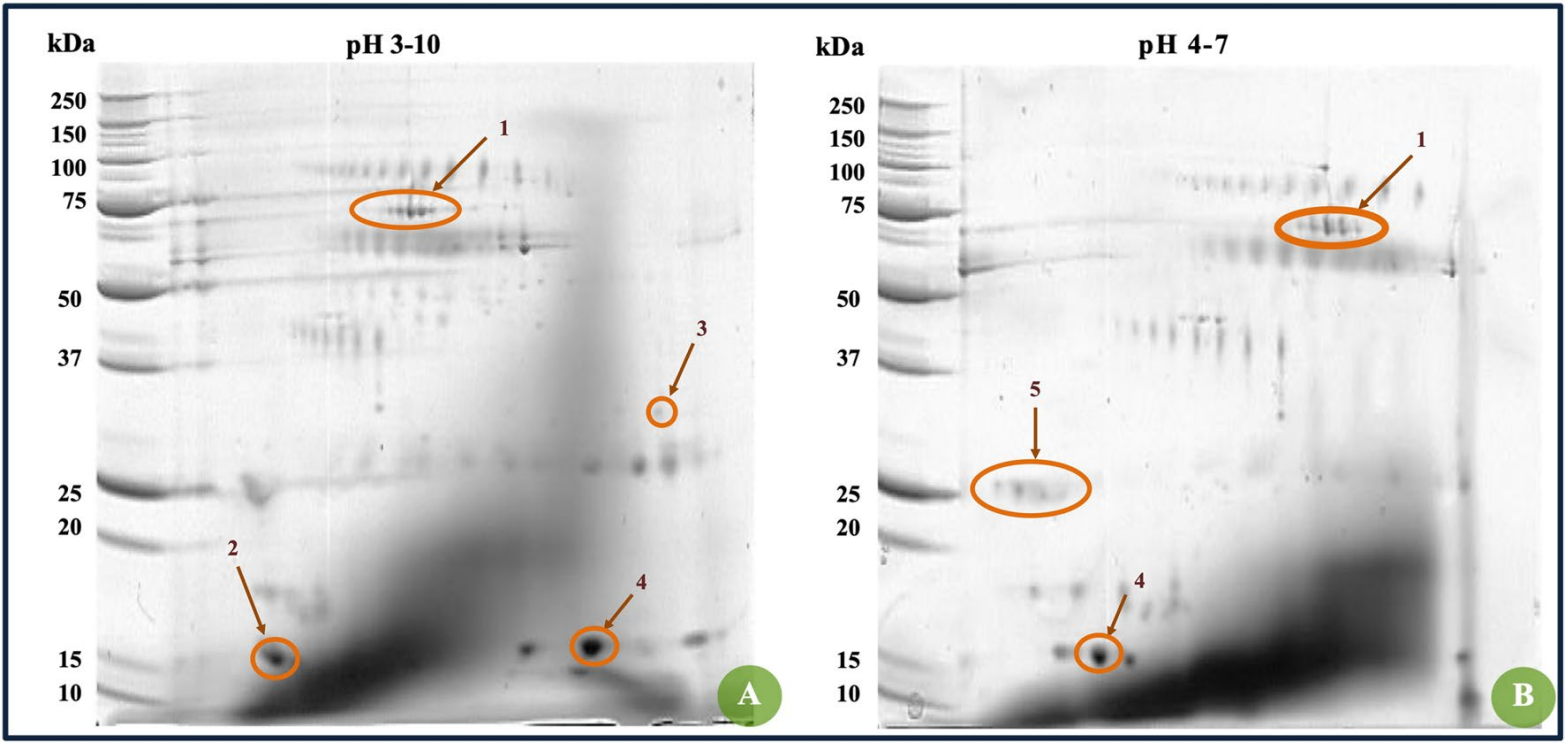
Reference 2D gel map of human saliva proteins from the samples pooled. Proteins were initially separated by isoelectric focusing (pH 3–10 and 4–7) and in the second dimension on the orthogonal 12% SDS-polyacrylamide gel under reducing conditions. The spots were visualized by staining with Coomassie blue R-250. The arrows indicate the presence of certain proteins assumed to be different isoforms of salivary amylase (1), enamel film precursor cystatin SA-III (2), basic proline-rich phosphoprotein (PRP) (3), acidic PRP (4), and PRP (5) based solely on the electrophoretic migration pattern in accordance with previous reports [28–30]

Figure 1 indicates the electrophoretically verified integrity of the saliva samples collected. Once this was confirmed, a pool with a final concentration of 0.53 mg/mL was obtained from the saliva samples collected. This pool was then processed to obtain a reference 2-dimensional gel map of the proteins in the whole saliva pool (Fig. 2). All in vitro assays of salivary pellicle formation on the tested substrates (Ti and enamel) were conducted using the saliva pool.

### Analysis of the salivary pellicle formed in vitro on Ti and enamel surfaces

**Fig. 3:**
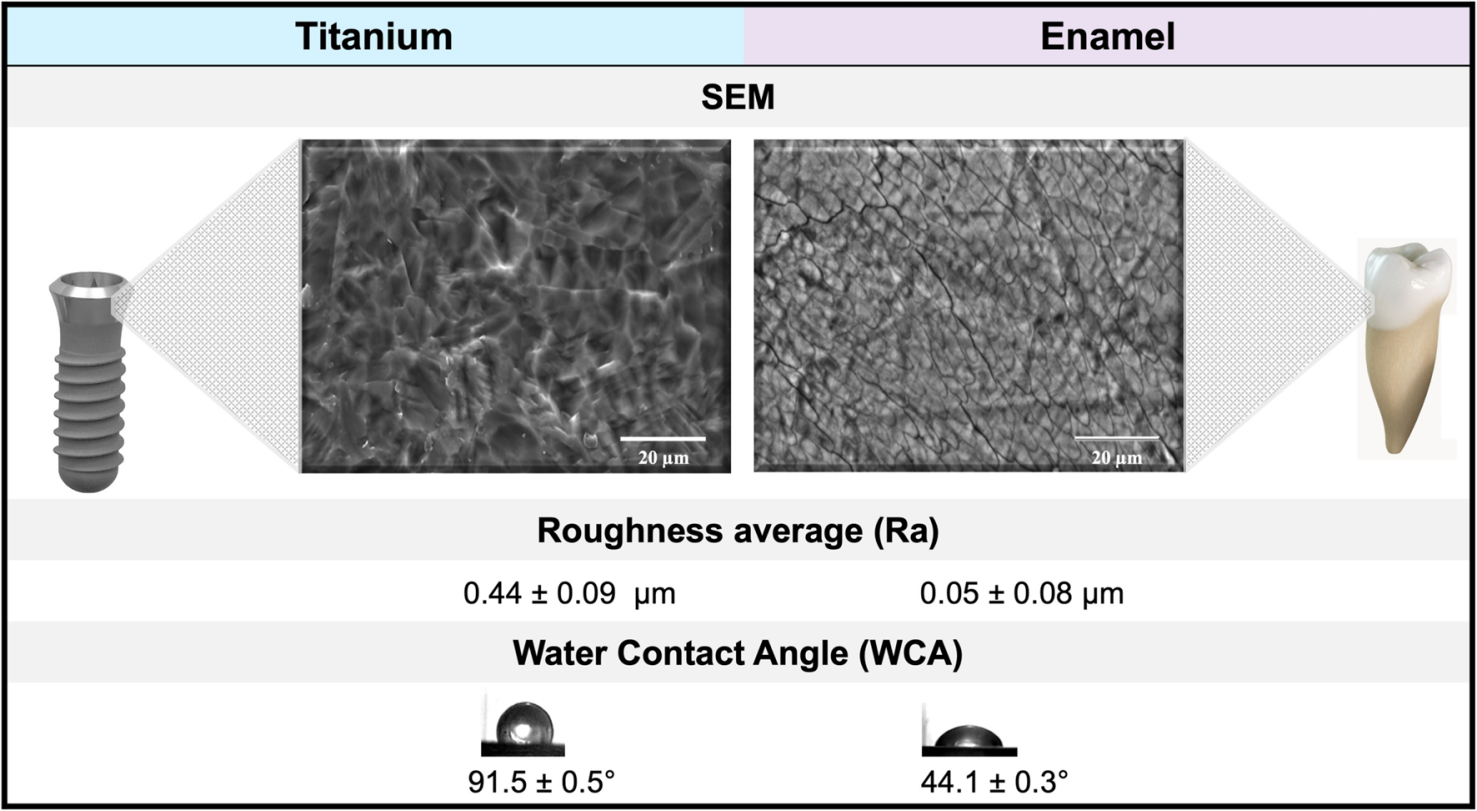
A description of the primary characteristics (topography, Ra, and WCA) of the surfaces used for all tests, salivary pellicle formation, and biofilm development, is presented. Titanium surfaces are representative of the implant neck, while enamel surfaces were used as a biological surface reference

Figure 3 shows the general characteristics of the titanium and enamel surfaces used for all assays, pellicle formation, and biofilm development.

**Fig. 4:**
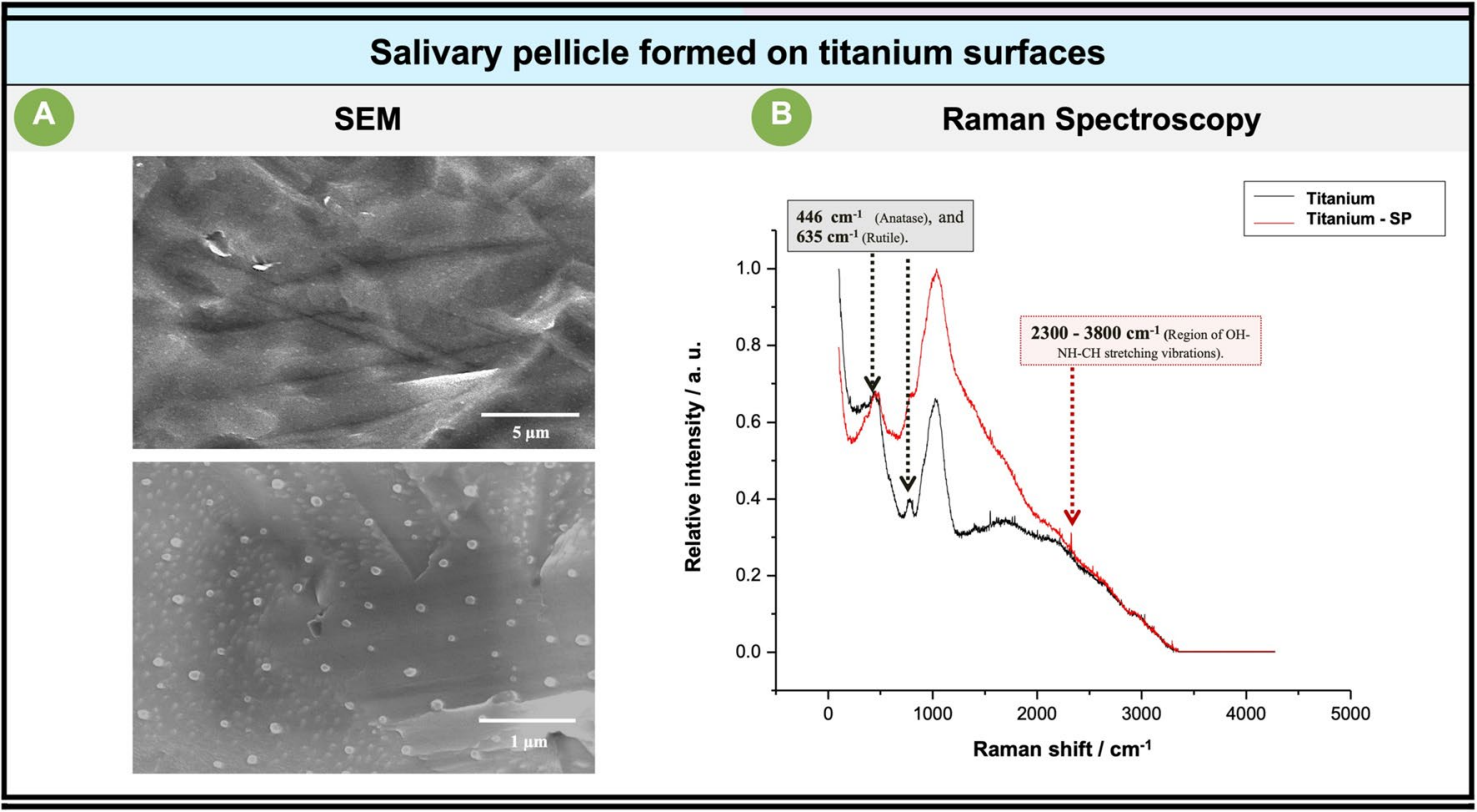
The images on the left (**A**) show the representative micrographs of the titanium surface covered by the salivary pellicle. On the right (**B**) Raman spectrum of the titanium surface (black spectrum), and the Raman spectrum of the titanium surface covered by the salivary pellicle (red spectrum)

**Fig. 5:**
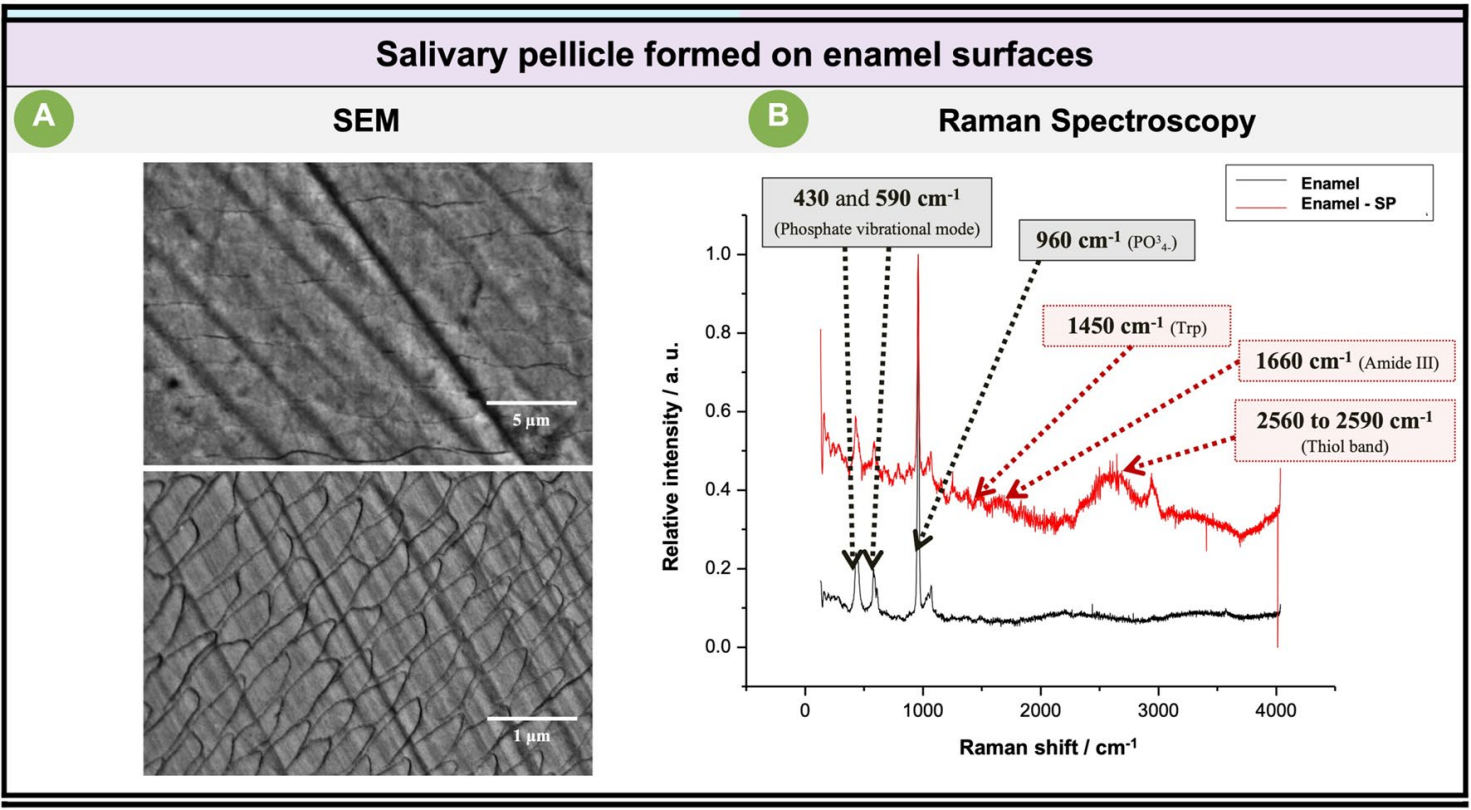
The images on the left (**A**) show representative micrographs of the enamel surfaces covered by the salivary pellicle. On the right (**B**) Raman spectrum of the enamel surface (black spectrum), and the Raman spectrum of the enamel surface covered by the salivary pellicle (red spectrum)

After incubating the tested substrates (Ti and enamel) with the saliva pool, the salivary pellicle formed under in vitro conditions was confirmed by SEM analysis and Raman spectroscopy (Figs. 4 and 5).

The previous figure shows a SEM view of the titanium surfaces (Fig. 4A) incubated with the saliva pool showing the presence of microglobules periodically coating the surface. To corroborate that the formation of globules periodically coating the salivary film formed on the Ti surfaces also occurred under in situ conditions, Ti and enamel surfaces were incubated intraorally to induce the formation of an immature (3 min of intraoral incubation) and mature (120 min of intraoral incubation) salivary layer. This test confirmed the presence of a bacteria-free electrodense layer with periodically distributed globular formations covering the titanium surfaces, but not the enamel surfaces, by TEM analysis (Supplementary data).

The Raman spectroscopy analysis confirmed that these microglobules corresponded to the salivary pellicle formed on the titanium surfaces. In this regard, it is important to emphasize that titanium metal has an inherent property of forming a thin amorphous or poorly crystallized oxide layer on its surface; this oxide layer can exist in three main polymorphs: anatase, rutile, and brookite [31]. Titanium oxide (TiO_2_) phases are usually Raman active in the 100–900-cm^−1^ region [32–34]. According to this, the Raman analysis performed on the control titanium surfaces (the ones uncovered by the salivary film) peaks at 446.6 and 635 cm^−1^ (black spectrum) characteristics of rutile [35] and anatase [36] phase TiO_2_, respectively, identified, indicating that the rutile and anatase TiO_2_ crystallites were the major species on the tested titanium substrates.

The Raman analysis done on the titanium surfaces covered by the salivary pellicle revealed subtle changes in the Raman spectrum (Fig. 4B), identifying primarily a peak between 2300–3800 cm^−1^ (red spectrum) which could correspond to the region of the OH-NH-CH stretching vibrations [37], confirming the presence of organic material (salivary pellicle) covering the tested titanium surfaces.

In contrast to what is observed in the titanium surfaces, enamel surfaces did not exhibit evidence of salivary pellicle formation (SEM micrographs above) (Fig. 5). However, according to the Raman spectroscopy (red spectrum), one of the regions that confirmed the formation of the salivary pellicle on the enamel surfaces is observed between 1700 and 2850 cm^−1^; this region contains only one major band of interest in natural amino acids and proteins, the thiol SH stretching, at 2560 to 2590 cm^−1^ [38]. Another confirmation of the salivary pellicle formed on the enamel surfaces corresponds with the peaks at 1660 cm^−1^ and ~1450 cm^−1^, which correspond with an amide III and tryptophan, respectively [38]; these bands are related to various glycoproteins that are known to be constituents of saliva, especially mucin matrices [39], and therefore part of the salivary pellicle formed.

Furthermore, in the Raman spectroscopy of the enamel surfaces not covered by the salivary pellicle, it is possible to observe a dominant peak at 960 cm^−1^. In literature, this peak is assigned to a PO_4_^3−^ vibration [40], thus confirming that the surface analyzed consisted of natural enamel (black spectrum) (Fig. 5).

### Evaluation of the effect of saliva pellicle on titanium and enamel surfaces on forming a biofilm of *S. gordonii*, *A. naeslundii*, *F. nucleatum* subsp *nucleatum*, *and P. gingivalis*

**Fig. 6:**
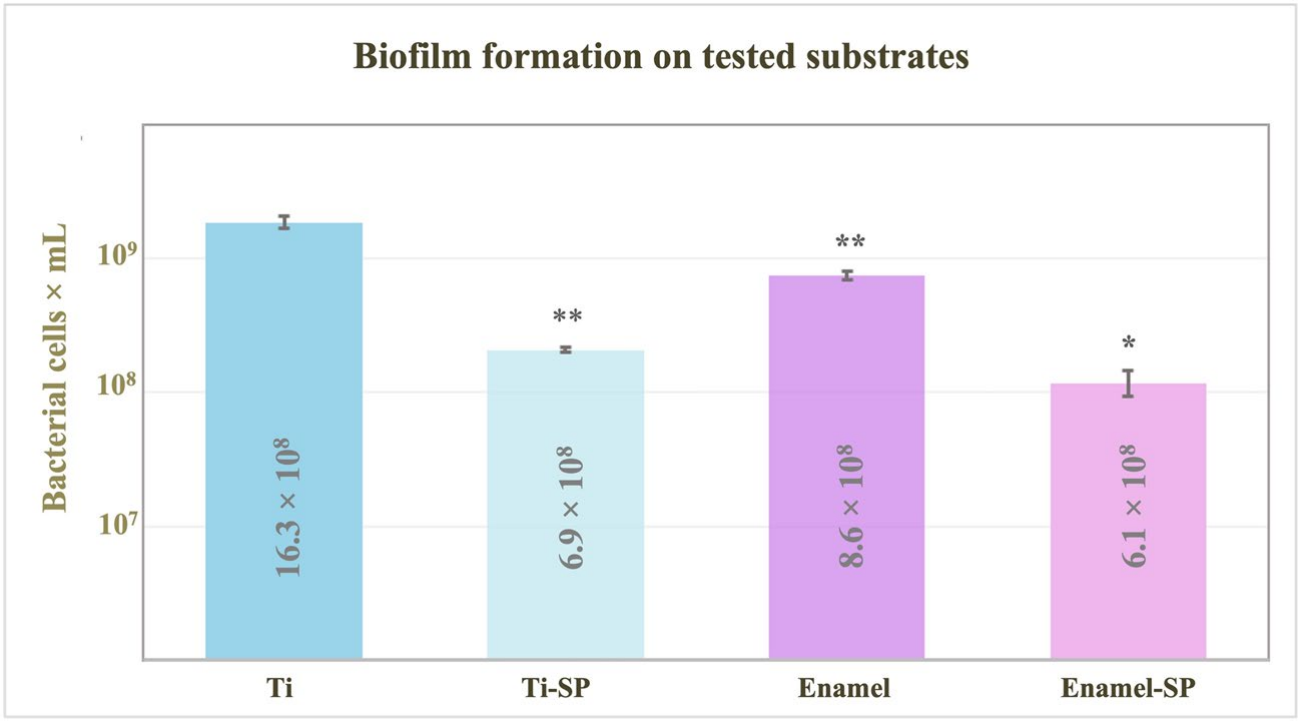
Total bacterial counts (bacterial cells × 10^8^/mL) after 12 h of anaerobic incubation on titanium and enamel surfaces, covered (Ti-SP and Enamel-SP) or not covered (Ti and Enamel) by the salivary pellicle. Values are presented as the mean ± EEM. *p < 0.05, **p < 0.01 vs Ti surfaces

The following results demonstrate the potential of the salivary pellicle formed on titanium and enamel substrates to modulate the formation of a biofilm composed of *S.* and *A. naeslundii*, *F. nucleatum* subsp. *nucleatum*, and *P. gingivalis* (Fig. 6).

The presence of the salivary pellicle directly influenced biofilm formation on titanium and enamel surfaces. In general, it was observed that on the substrates not covered by the salivary pellicle, higher bacterial cell counts were detected compared to surfaces covered by the salivary pellicle (Ti-SP and enamel-SP). Notably, biofilm formation was significantly higher on titanium surfaces, the substrates with higher roughness and contact angle, compared to enamel, the surfaces with lower roughness and contact angle.

On titanium surfaces covered by the salivary pellicle, a 2.3-fold reduction in bacterial cell counts (from 16.3 × 10^8^ cells/mL to 6.95 × 10^8^ cells/mL) was observed when compared to titanium surfaces not covered by the SP (*p* < 0.01). The same was observed on enamel surfaces, a 1.4-fold reduction in bacterial cell counts, from 8.61 × 10^8^ cells/mL to 6.19 × 10^8^ cells/mL, was quantified on SP-covered enamel substrates compared to those that are not covered by the protein pellicle (*p* <0.05). Notably, no statistically significant differences were observed between the pellicle-covered surfaces (Ti-SP and enamel-SP) and the number of bacterial cells adhered to them (*p* = NS).

**Fig. 7:**
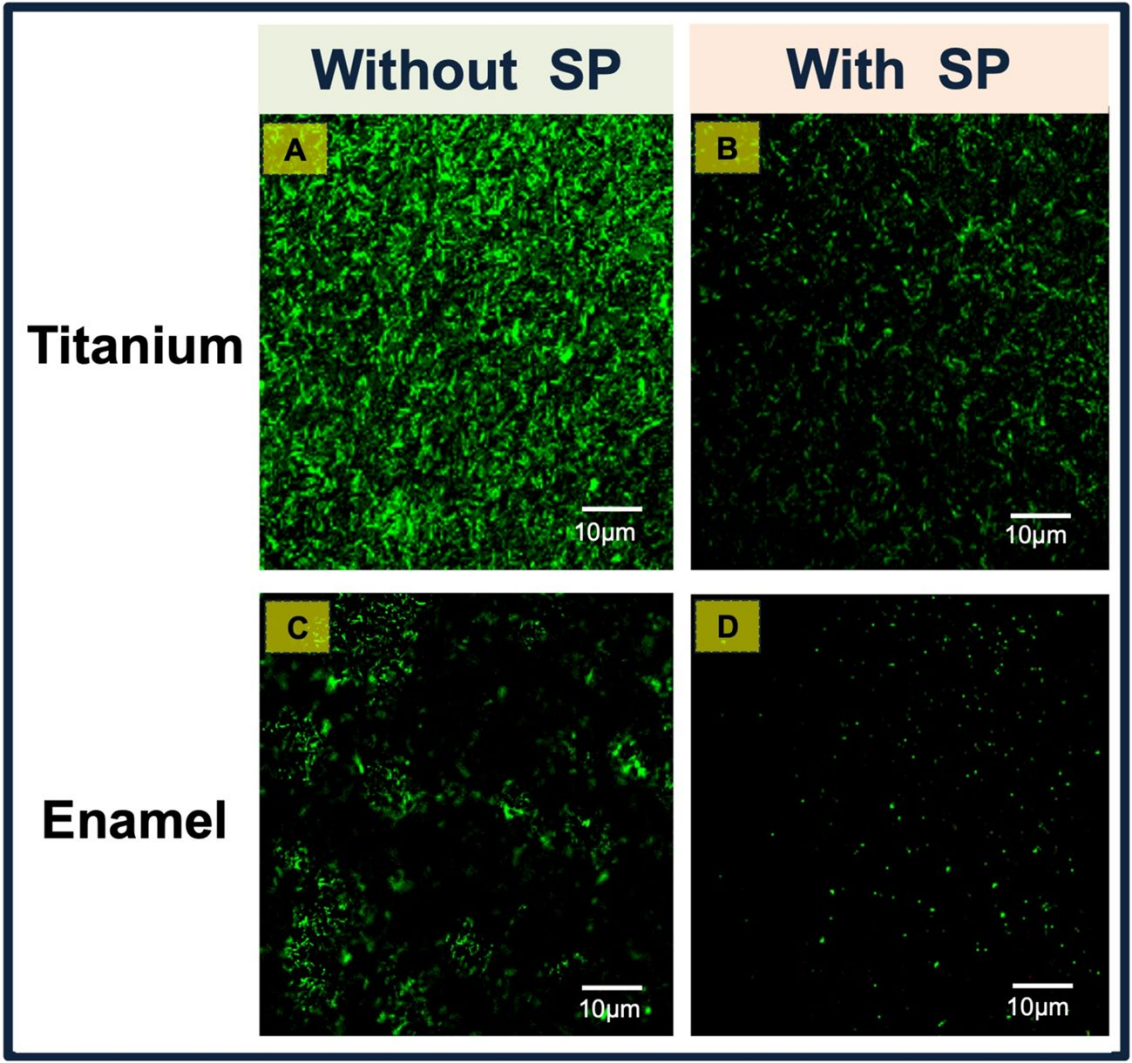
Fluorescence microscope images of biofilm formation after 12 h of culture at 37 °C under anaerobiosis conditions on titanium and enamel substrates, without salivary pellicle (**A** and **C**, respectively), and the surfaces covered by the salivary pellicle (**B** and **D**, respectively). The images represent the microscopic fluorescent visualization of the adherent bacteria after the adoption of the live/dead staining. More bacteria are seen on the uncoated surfaces (control) compared to the salivary pellicle–coated surfaces. Scale bar 10 μm

Figure 7 shows representative fluorescence images of biofilm formation at 12 h on titanium and enamel surfaces, not covered and covered by the salivary pellicle. As can be seen, most of the bacterial cells were stained green, assuming that the bacterial strains were alive at the time of the observations. Notably, more bacteria can be seen on biofilms formed on the substrates with higher roughness and contact angle (titanium), regardless of whether the salivary pellicle coats them. In comparison, it could be confirmed that the presence of salivary pellicles on titanium and enamel substrates caused a reduction in the number of adherent bacterial cells (Fig. 7B and D).

## Discussion

As certain surfaces of dental implants are exposed to the oral environment, they are coated with plasma and salivary proteins [7], which will allow microbial adhesion that ultimately led to biofilm formation. The formation of complex oral biofilms has been recognized as one of the leading causes of dental implant failure [41]. Due to the above, the present work aims to explore the ability of the salivary pellicle (SP) formed on titanium surfaces to modulate the development of oral biofilms and compare this phenomenon on a biological reference such as enamel surfaces.

It has been reported that SP formation on natural enamel surfaces reaches maturation within 90 to 120 min [42–44]. In the present study, the formation of a salivary pellicle on Ti and enamel surfaces after they were incubated for 2 h with the saliva pool was confirmed. Specifically, using SEM microscopy on Ti surfaces, it was possible to observe periodically distributed globular formations on the salivary pellicle. The observation of globule-like structures in the salivary pellicle formed on Ti surfaces is in agreement with previous reports indicating that the salivary layer formed in situ for periods of 30–120 min on biomaterial surfaces reveals a complex globular surface texture with diameters of the adsorbed globule clusters varying from 80 to 200 nm [45, 46]. Since the first elucidation of the ultrastructural pattern of the salivary film formed on titanium surfaces in the 1990s [7, 47], the present study represents the first report on the morphogenesis of the salivary film formed in vitro and in vivo on Ti substrates representative of the neck of dental implants in clinical use.

The pellicle layer formed on the Ti substrates evaluated here showed distinct differences in ultrastructural appearance compared to reported previously [7, 47]. In this regard, transmission electron microscopic analysis showed that the Ti substrates were covered by a thin continuous 5–10 nm layer of high electron density, both in the specimens incubated for 3 min and for 2 h. On top of this thin layer, in the salivary pellicle formed at 2 h, a second heterogeneous layer of lower density of about 30 nm was formed, on top of which it was possible to observe finely delimited 50–100-nm globular formations distributed periodically with ~1.5 μm spacings, this finding was directly confirmed by the SEM images. Therefore, the salivary pellicle’s ultrastructure may reflect not only the local conditions [7, 47] but also material-dependent characteristics.

Like salivary pellicle formation, biofilm formation on oral surfaces is a highly selective process. According to different studies, initial bacterial adhesion to metallic biomaterial surfaces is directly influenced by the physical–chemical features of the substrates, like topography, roughness, and hydrophobicity, among others [48, 49]. However, there is increasing evidence that bacterial adhesion is mainly influenced by the salivary pellicle formed on the surface of the biomaterials once the substrate is exposed to the oral environment [50–52]. Consequently, it is vital to consider the salivary pellicle as a determining factor in the biomaterial–oral ecosystem interrelations when studying bacterial adhesion and the consequent formation of biofilms.

In the oral cavity, the initial colonization of microorganisms occurs with the maturation of the salivary pellicle [42], which provides the specific receptors for bacteria to adhere [53]. Initial colonizers on enamel surfaces are usually *Streptococcus* and *Actinomyces* species [54–56]; specifically, adhered *Streptococcus* release glycosyltransferase into the biofilms extracellular matrix, which helps to enhance the colonization of other species like *F. nucleatum* or *P. gingivalis* [55, 57].

In this report, it was found that salivary pellicles formed on the studied substrates modulated the formation of biofilms consisting of *S. gordonii*, *A. naeslundii*, and the pathobionts *F. nucleatum* and *P. gingivalis* by decreasing their colonization on the Ti and enamel surfaces in comparison to the results observed on substrates without the salivary pellicle coating. Using nano-LC-MS/MS, a previous study conducted by our group has shown that the salivary pellicle formed on Ti surfaces, similar to those used in this study, can contain over 240 different proteins [20]. Therefore, the presence of proteins such as α-amylase, histatin 5, and cystatin S in the salivary layer formed on the surfaces is anticipated and may be the responsible for inducing the significant reduction in the adhesion of the oral bacteria tested here. Previously, the aforementioned proteins have been reported to have a significant antibacterial effect when adsorbed on titanium surfaces [26, 58].

Although the present work did not show a decrease in individual bacterial species, but rather a decrease in total biofilm formation on titanium and enamel surfaces, it is to be expected that some decrease in *P. gingivalis* load had occurred. Importantly, implant surfaces colonized by high levels of *P. gingivalis* are associated with the development of peri-implantitis.

Finally, the modulating effect of the salivary pellicle was clearly confirmed when the biofilm formation was examined on titanium and enamel surfaces in the absence of salivary coverage. Under such experimental conditions, the bacterial adhesion patterns observed were more dependent on the physicochemical properties of the surfaces evaluated. Accordingly, the higher roughness and hydrophobicity of the Ti surfaces were correlated with higher levels of bacterial adhesion. Rough surfaces have been demonstrated to provide greater contact areas and, therefore, more potential bacterial binding sites than smooth or less rough surfaces [59].

## Conclusions

The present study confirmed the formation of a 2-h salivary pellicle on titanium surfaces and the biological reference dental enamel by TEM, SEM, and Raman spectroscopy; this represents the first attempt to use a spectroscopic tool to investigate the salivary pellicle formed on Ti surfaces. Besides, salivary pellicles formed on titanium and enamel substrates were able to regulate the development of representative oral biofilms, modulating bacterial adhesion over physicochemical features, such as roughness and hydrophobicity of the substrates.

Considering the significance of the salivary pellicle to modulate the oral biofilm formation on Ti surfaces used in the current dental implantology, future studies need to examine salivary pellicle formation using saliva samples from subjects with hyposalivation or periodontitis to determine if any changes in the modulatory effect of the pellicle layer formed from the saliva of such populations adversely affect biofilm formation.

### Supplementary information

ESM 1 (DOCX 2096 kb)

## Data Availability

The data that support the findings of this study are available from the corresponding author upon reasonable request.
