# Supplementary material for: Salivary pellicle modulates biofilm formation on titanium surfaces
Source: Clin Oral Investig. 2023 Aug 30;27(10):6135–45. doi: 10.1007/s00784-023-05230-9 (PMC10560156; doi:10.1007/s00784-023-05230-9)
Supplement: Supplementary file 1 — (DOCX 2096 kb) [file 784_2023_5230_MOESM1_ESM.docx]

**Supplementary information.**

***Transmission electron microscopy evaluation of the in situ salivary pellicle formation on titanium and enamel substrates***

To corroborate whether the formation of microgobules in the salivary film formed in vitro on the titanium surfaces observed by scanning microscopy also occurred under in situ conditions, the ultrastructure of the salivary films formed in situ on the tested surfaces was explored by TEM. In brief, transparent custom-made acrylic splints were fabricated as a carrier of the Ti and enamel specimens, which were fixed in the left and right buccal positions, in the molar and premolar regions. Silicon impression material (President Light Body®, Colténe, Altstaetten, Switzerland) was used to fix the Ti and enamel specimens to the splints. The splints were exposed intraorally over periods of 3 min or 2 h.

After the salivary pellicle formation, the surfaces were removed from the splints and individually rinsed with deionized water from a pressure cylinder to gently remove residual saliva and non-adsorbed epithelial cells. Then, the specimens were fixed with a solution consisting of 1% glutaraldehyde and 1% paraformaldehyde in 0.1 M cacodylate buffer for 60 min at 4 °C. The samples were then washed five times for 10 min with 0.1 M cacodylate buffer and stored in the last buffer solution at 4 °C. For visualization of the organic pellicle structure, samples were post-fixed with 2% osmium tetroxide in cacodylate buffer for 2 h. Afterward, the specimens were washed five times with distilled H_2_O and dehydrated using increasing concentrations of ethanol. Subsequently, the samples were incubated twice in acetone for 20 min and incubated overnight in a mixture of Araldite CY212 (Agar Scientific, Stansted, UK) and acetone (1:1). Before ultrathin sectioning, the substrate material was removed as previously described by Hannig, M. [1]. Ultrathin sections of the tested samples were cut in series with an ultramicrotome (Ultracut E, Reichert, Bensheim, Germany) using a diamond knife (Microstar 45°, Plano, Wetzlar, Germany). Characteristic micrographs were taken at magnifications varying from 2.500- to 80.000-fold by a TEM Tecnai12 Biotwin (FEI, Eindhoven, Netherlands). The TEM micrographs were taken at 30 000 and 50 000-fold magnification.

The following figure shows representative micrographs of the salivary pellicle formed *in situ* at 3 min and 2 hrs on the titanium and enamel substrates.

**
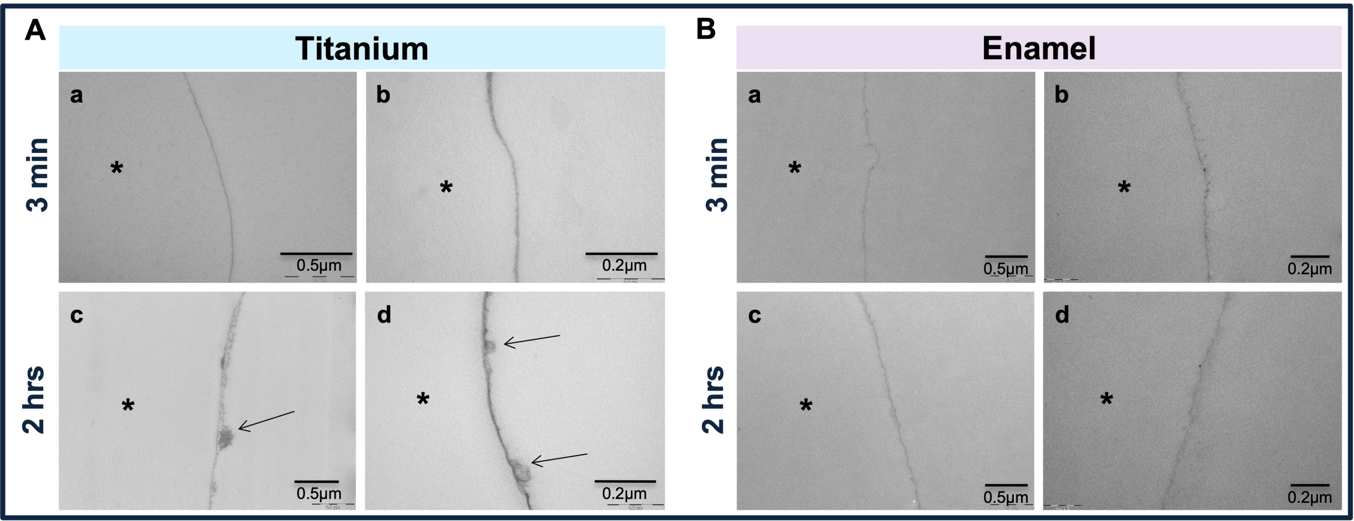
Figure.** Transmission electron microscopy (TEM) micrographs of the salivary pellicle formed *in situ* on the Ti (**A**) and enamel (**B**) surfaces positioned intraorally on the buccal surfaces of the upper molar area after exposure for 3 min (a and b) and 2 h (c and d) periods. Ti and Enamel surfaces were removed during the processing of specimens for TEM analysis (*Previous Ti/enamel surface).

Figure **Ac, d** confirmed the presence of globular clusters of salivary polymers (arrows) periodically covering the 2-hour salivary film formed *in situ* on the titanium substrates. These globular clusters averaged between 50-100 nm.

1. Hannig M (1997) Transmission electron microscopic study of in vivo pellicle formation on dental restorative materials. Eur J Oral Sci 105:422-33. doi: 10.1111/j.1600-0722.1997.tb02139.x
